# Dual implication of endothelial adhesion molecules in tumor progression and cancer immunity

**DOI:** 10.1080/19336918.2025.2472308

**Published:** 2025-03-12

**Authors:** Louis-Emmanuel Chriqui, Sabrina Cavin, Jean Yannis Perentes

**Affiliations:** a Division of Thoracic Surgery, Department of Surgery, CHUV, Lausanne University Hospital, Lausanne, Switzerland; b Agora Cancer Research Center Lausanne, Lausanne, Switzerland

**Keywords:** Adhesion molecules, ICAM, immunotherapy, metastasis, selectins, VCAM

## Abstract

Adhesion molecules are proteins expressed at the surface of various cell types. Their main contribution to immunity is to allow the infiltration of immune cells in an inflamed site. In cancer, adhesion molecules have been shown to promote tumor dissemination favoring the development of metastasis. While adhesion molecule inhibition approaches were unsuccessful for cancer control, their importance for the generation of an immune response alone or in combination with immunotherapies has gained interest over the past years. Currently, the balance of adhesion molecules for tumor promotion/inhibition is unclear. Here we review the role of selectins, intercellular adhesion molecules (ICAM) and vascular cell adhesion molecules (VCAM) from the perspective of the dual contribution of adhesion molecules in tumor progression and immunity.

## Introduction

Vascular endothelium endorses various functions owing to its structural role in separating blood from tissues. Endothelial cells (ECs), which cover the inner side of vessels, are responsible for vascular homeostasis and tone. In addition to vascular homeostasis [1,2], ECs are important for the innate immunity through the expression of Toll-Like receptors which contribute to the recruitment of immune cells to a target organ. Additionally, the upregulation of adhesion molecules at the surface of ECs supports the inflammatory state through leukocyte trafficking [3].

Adhesion molecules are transmembrane glycoproteins that contribute to the recruitment of circulating leukocytes from the blood to tissue. Immune cell ligands bind to the adhesion molecules which is followed by a cascade of interactions ultimately causing the extravasation of circulating immune cells [4,5].

Given their critical regulatory function between systemic circulation and tissues, EC and their adhesion molecules play a central role in cancer. While the tumor promoting effect of adhesion molecules through dissemination of cancer cells was well described, the capacity of these molecules to reprogram the tumor microenvironment (TME) and generate an immune response directed against tumors has created interest over the recent years, particularly with the development of immunotherapies [6–8]. Interestingly, the balance between tumor promotion (metastasis spread) and tumor inhibition (immunity development) is currently not established. Here we propose to review the dual implication of various endothelial adhesion molecules for cancer promotion/inhibition.

## Overview of adhesion molecules: selectins, ICAM and VCAM

### Selectins

Selectins represent a first group of cell adhesion molecules composed of three different glycoproteins, expressed either at the surface of endothelial cells (E-Selectin), leukocytes (L-Selectin) or platelets (P-Selectin) [9–12]. All three mediate the rolling of blood cells at the endothelial surface thus initiating leukocyte attachment to platelets, endothelial cells, and other leukocytes at sites of tissue injury and/or inflammation [13–15]. All the three members of the selectin family are type I transmembrane protein exhibiting an N-terminal lectin-like domain, an epidermal growth factor (EGF) domain and a variable short consensus repeat (CRP) domain [13]. Ligands binding to these molecules are numerous and diverse. Most of them carry sialylated, sulfated and/or fucosylated sequences [16]. The expression of E-Selectin is triggered by various inflammatory cytokines such as IL-1, TNF-α or bacterial endotoxin as a consequence of endothelial activation [17,18]. E-Selectin contributes to the recruitment of various immune populations including neutrophils, monocytes and T lymphocytes [9,19–21]. E-Selectin recognizes sialofucosylated lactosaminyl tetrasaccharides, prototypically sialyl Lewis x (sLex) and its structural isomer sialyl Lewis a (sLea) and binds to it in a calcium dependent manner [22].

P-Selectin is stored in the α-granules of platelets and Weibel-Palade bodies of endothelial cells. Following stimulation, notably by thrombin, P-Selectin is rapidly translocated at the surface of platelets or endothelial cells [23,24]. Ligands of P-Selectin – sLeX and P – selectin glycoprotein ligand 1 (PSGL–1) – are expressed at the surface of numerous immune cells including neutrophils and monocytes, thus supporting their adhesion to platelets or endothelial cells [14,15,25].

L-Selectin is constitutively expressed on most of immune cells including neutrophils, monocytes and lymphocytes. Its function is decreased through a down-regulation in its gene transcription, thus precluding its expression at the surface of the cells [26,27]. It interacts with sialomucin ligands either on vascular or tumoral compartment [28]. In addition to the adhesion to endothelium, L-Selectin holds an important role in leukocytes homing by allowing migration to lymph nodes [29,30].

### Intercellular adhesion molecules

Intercellular adhesion molecules (ICAM) are a second group of adhesion molecules expressed at the surface of endothelial cells. The family is composed of five structurally related members, ICAM-1 to ICAM-5 characterized by the presence of extracellular immunoglobulin domains [31–33]. Preferred ligands for ICAM are a subgroup of integrins called β2-integrins. This subgroup is composed of four different integrins: CD11a/CD18 or LFA-1; CD11b/CD18 or Mac-1 or CR3; CD11c/CD18, or p150.95 or CR4; and CD11d/CD18. Each exhibits various expression pattern and binding capacities. For example, while LFA-1 is expressed only on leukocytes and binds only specific molecules close to ICAM, Mac-1 is expressed on myeloid cells and can bind up to 40 different molecules [34–36]. ICAM-1 is expressed at low levels on many cell types including epithelial cells, endothelial cells and immune cells [37]. In addition to contribution in homeostasis and injury repair, ICAM-1 is implicated in different steps of leukocytes trans-endothelial migration from rolling to adhesion to the endothelium mainly through the binding of its most reported ligand, LFA-1 [38–41]. Moreover, ICAM-1 is also an important contributor of the immunological synapse between antigen presenting cells and effector T-cells. While its role is not clearly understood, the dynamic expression of the molecule has been reported to be implicated in the priming of T-cells [42,43].

Contrarily to ICAM-1, with an expression closely dependent of inflammatory cytokines such as IL-1 or TNF-α, ICAM-2 is constitutively expressed at the surface of endothelial cells [44–47]. In addition to the leukocyte trafficking regulation role, ICAM-2 has also been reported to play a role in angiogenesis and in the control of the endothelial cell junction and barrier function [48–51]. Identified ligands for ICAM-2 are LFA-1 and DC-SIGN [52,53].

ICAM-3 is constitutively expressed at the surface of resting T lymphocytes and possesses a co-stimulatory activity in T lymphocytes. In addition, ICAM-3 is also implicated in cell contacts through its ability to induce LFA-1–ICAM-1 adhesion [54–59].

The expression of ICAM-4 is specific for erythrocytes [60]. In addition to the interaction with leukocytes and myeloid cells through CD11a/CD18 and CD11b/18 respectively, ICAM-4 can bind to αv integrins (αvβ1, αvβ3, and αvβ5) on non-hemopoietic cells, α4β1 on hemopoietic cells, and αIIbβ3 on platelets [61–64]. It has been reported that ICAM-4 could mediate interactions between red blood cells and macrophages [65]. Thus, ICAM-4 plays a role in cell interaction implicated in blood cells regulation, thrombosis and hemostasis.

ICAM-5 is the largest ICAM molecule, exhibiting nine extracellular immunoglobulin domains and is confined to the central nervous system with a surface expression on specific neurons [66]. ICAM-5 is acting at the interface of nervous and immune system. These interactions could result in microglia morphology shaping, regulation of synapses or cytokine release in the central nervous system [67–72].

### Vascular cell adhesion molecule I

**Figure 1.**
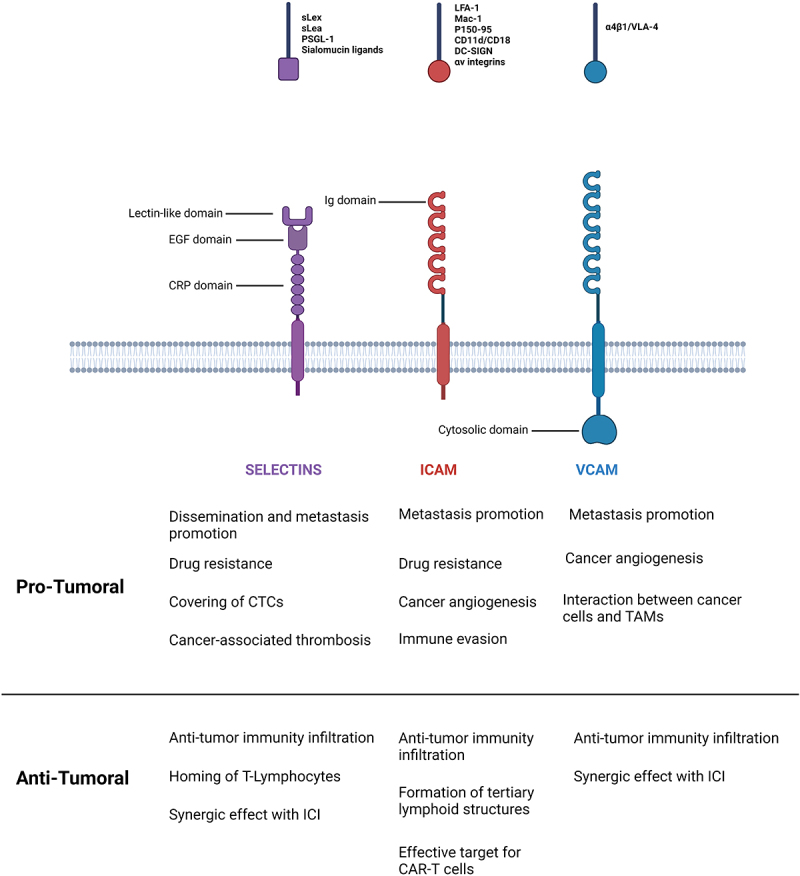
Structure of selectins, ICAM and VCAM adhesion molecules and their main respective ligands. Pro-tumoral and anti-tumoral roles are indicated. sLex: sialyl Lewis x, sLea: sialyl Lewis a, PSGL-1: P–selectin glycoprotein ligand 1, ig: immunoglobulin, EGF: epidermal growth factor; CRP: consensus repeat, CTCs: circulating tumor cells, ICI: immune checkpoints inhibitors, TAMs: tumor associated macrophages.

Vascular cell adhesion molecule I (VCAM-1) is a glycoprotein containing an extracellular domain with six or seven immunoglobulin (Ig)-like domains, a transmembrane domain, and a cytoplasmic domain [73]. VCAM-1 is mainly expressed on endothelial cells following pro-inflammatory cytokines such as TNF-α or exposition to reactive oxygen species (ROS). However, in condition of high and sustained inflammation, its expression has been reported on additional cell types including tissue macrophages, dendritic cells, bone marrow fibroblasts, myoblasts, oocytes, Kupffer cells, Sertoli cells, and cancer cells [74–77]. By binding to its leukocyte ligand α4β1/VLA-4 integrin, VCAM-1 plays a key role in leucocyte recruitment through the adhesion of circulating immune cells to the endothelium and the activation of signaling pathways involved in trans-endothelial migration [78,79]. A summary of the structures of selectins, ICAMs and VCAMs is reported in Figure 1.

## Contributions of endothelial adhesion molecules to tumor progression

Besides their pro-immune function, adhesion molecules have also been implicated in cancer progression through the promotion of metastasis.

### Selectins

Tumors cells that disseminate from their primary location, do so by intravasating into the systemic circulation and extravasating into target metastasis organ sites. This process involves a cascade of distinct endothelial interaction steps. This is favored by the upregulated expression of selectin ligands (such as sLex, sLea, CD34 or MAdCAM-1) at the surface of cancer cells [80]. Thus, the extravasation of tumor cells at distant organs was shown to depend on the interaction of endothelial E-Selectin and tumor selectin ligands [81]. Consistently, high levels of selectin expression in cancer was shown to correlate with poor prognosis [82–84]. Recently, Tanio et al. [85] showed that the expression of E-Selectin ligands at the surface of clear cell renal cell carcinoma was a strong prognostic biomarker in patients. In hematologic cancers, E-Selectin expression at the surface of leukemic blasts was also associated with a worse prognosis [86,87]. Given the contribution of E-Selectin in metastasis, studies have tried to inhibit its expression to limit cancer dissemination. Brodt et al. [88] showed in a model of highly metastatic colon cancer that endothelial E-Selectin depletion significantly decreased the dissemination of colon cancer to the liver. Lange et al. [89] showed Bortezomib precluded the cytokine dependent expression of endothelial E-Selectin, thus impairing spontaneous lung metastasis *in vivo*. Interestingly, while ICAM-1 and VCAM-1 were depleted in presence of Bortezomib, authors concluded loss of E-Selectin alone was necessary to reduce adhesion of tumor cells. Khan et al. [90] observed a decrease in triple negative breast cancer metastasis in lungs following depletion of lung E, P and L-selectins. In an in vivo model of melanoma, Coppo et al. [91] confirmed that high levels of endothelial E-Selectin expression correlated with increased adhesion of tumor cells while inhibition with cimetidine was able to diminish tumor cell dissemination. In addition to its effect on metastasis, endothelial E-selectin is known to induce drug resistance [92]. In acute myeloid leukemia engrafted mice, E-selectin expressed by cancer cells was associated with a chemoresistance promoted through the Wnt pathway [93]. It has also been reported that PI3K/AKT/NF-κB pathway was an important mediator of chemoresistance induced by E-Selectin at the surface of tumor cells [94]. In solid cancer, Morita et al [95] showed that inhibition of vascular E-Selectin enhanced the anti-tumor effect of Doxorubicin and reduced tumor infiltration of pro-tumoral macrophage M2.

P-Selectin expression has been reported to promote cancer dissemination. By binding P-Selectin, circulating tumor cells benefit from the cover of platelets protecting them from shear forces and immune cells [96]. This pro-tumoral effect of P-Selectin and mucin/non-mucin-type glycoprotein have been well described in the past [97–101]. Kim et al. [97] investigated the contribution of P-Selectin in cancer progression and dissemination. They showed P-selectin-deficient mice harbored a slower growth of subcutaneous tumors and generated fewer lung metastases compared to control. Borsig et al. first described the contribution of P-Selectin to the dissemination of human carcinomas in immunodeficient mice [98] and later exposed its role in mediating the interactions between tumor cells and platelets in a murine adenocarcinoma in syngeneic immunocompetent mice [99]. Recent studies have also supported the contribution of P-Selectin to tumor progression: Cariello et al [102] showed in a colon cancer model that ablation of P-selectin in platelets significantly reduced tumor growth. Studying T cell lymphoma, Pereira et al. [103] found expression of the P-selectin ligand, PSGL-1, by the tumor cells was implicated in the development and dissemination of the cancer in different organs. The role of PSGL-1 was also investigated by Azab et al. [104] which drew the same conclusion in multiple myeloma. Zhang et al. [105] highlighted the preferred aggregation of low differentiated aggressive hepatocarcinoma cells with platelets through P-selectin. Conversely, abrogation of platelet aggregation with Clopidogrel attenuated platelet-tumor cell binding but also promoted hepatoma cell differentiation. Recently, the contribution of extracellular vesicles (EV) secreted by cancer cells in platelet aggregation through P-selectin expression have been investigated: Kim et al. [106] found that IL-8 released through cancer vesicles increased P-selectin expression at the surface of platelets and thus platelet aggregation. In addition, the level of platelet adhesion to vessel treated with cancer vesicles allowed to discriminate between breast cancer patients with and without metastasis. These results were supported by Gomes et al. [107] that showed aggressive breast cancer-derived EVs may contribute to cancer-associated thrombosis through an increase in platelet P-selectin exposure and platelet aggregation. This supports the implication of P-selectin in cancer-associated thrombosis previously described [108,109].

The contribution of L-Selectin to metastasis has been investigated in parallel to the contribution of E and/or P-selectin with similar implications [90,103,110]. Regarding the specific role of L-Selectin in cancer promotion, its expression in lymph node contributes to the dissemination of cancer cells in the lymphatic system [111]. Additionally, similarly to their interaction with platelets, tumor cells interact with circulating leucocytes by expressing L-Selectin ligands. These interactions favor survival of circulating tumor cells and the establishment of metastatic foci [112]. More specifically, research pointed out the critical role of myeloid cells in the dissemination of cancer cells through L-Selectin interaction. At the establishment of metastatic foci, an enhanced presence of CD11b-positive leukocytes associated with tumor cells was concomitantly detected, suggesting their involvement in this process [112]. Borsig et al. [99] observed a decrease in metastasis development in L-Selectin deficient mice. Interestingly, mice were deficient in T and B lymphocytes suggesting a specific contribution of neutrophils, monocytes, or NK cells. Läubli et al. [113] showed interactions between leucocytes and cancer cells through L-Selectin increased the production of CCL5 by endothelial cells. Inhibition of CCL5-dependent monocyte recruitment during the early phase of metastasis strongly reduced tumor cell dissemination. Evidence implicating lymphocytes in cancer cells migration remains poor. Head and neck squamous cancer cells has been shown to be able to bind to lymphocyte in presence of a shear stress similar to lymphatic flow through L-Selectin [114].

### ICAM

ICAM-1 contributes to cancer progression in different ways. As for other endothelial adhesion molecules, interactions with ICAM-1 on the surface of endothelium and its ligands expressed by the tumor cells favor tumor dissemination to secondary sites. This phenomenon has been highlighted as the suppression of ICAM-1 expression led to a decrease in cancer cell migration [115–117]. Chen et al also found that the expression of ICAM-1 by tumor cells was associated with a higher rate of bone metastasis and poorer prognosis in triple negative breast cancer [118]. This phenotype was partially explained by the ability of ICAM interactions to trigger the epithelial-to-mesenchymal transition program through TGF-β/SMAD. Taftaf et al investigated extensively the role of ICAM-1 at the surface of tumor cells in metastasis and found the molecule was involved in trans-endothelial migration but also in cluster formation of circulating tumor cells [119]. In a model of hepatocellular carcinoma, ICAM-1 was associated with increased vascular permeability through the VE-cadherin dependent interaction with endothelial cells [120]. In addition to its role in cancer dissemination, evidence suggest that ICAM-1 also plays a role in cancer angiogenesis. In triple negative breast cancer, Guo et al. [121] observed a reduction of vascular endothelial growth factor (VEGF) secretion in tumors of mice exposed to ICAM-1 inhibitors. Interestingly ICAM-1 expression levels in blood were reported as a reliable predictor of metastatic colorectal cancer response to bevacizumab (a VEGF receptor inhibitor) [122]. Similarly, in a clinical trial on non-small cell lung cancer (NSCLC), baseline plasma levels of ICAM-1 were found to be prognostic for survival and predictive of response to chemotherapy with or without bevacizumab: indeed, low baseline levels of ICAM-1 were associated with better survival and better response to bevacizumab [123]. More generally, ICAM-1 is reported as a prognostic cancer marker in oral cancer but also breast, colorectal and gastric cancer [124–127]. Pro-tumoral role of the other ICAM molecules are less investigated. ICAM-2 inhibition in the tumor promoted anti-tumor response in colon carcinomas mouse models [128]. A pro-tumoral role of ICAM-3 seems to be largely mediated through the PI3k/Akt pathway. Kim et al assessed the contribution of ICAM-3 in cancer cells for their proliferation through the PI3k/Akt pathway [129]. Using the same pathway and CREB pathway, ICAM-3 expression favors cancer invasiveness by upregulating expression of MMP-2 and MMP-9 [130]. In addition to cell proliferation and dissemination, activation of this pathway by ICAM-3 also promote anti-cancer drug resistance [131]. Finally, ICAM-3 expressed by tumor cells is also associated with a poor prognosis either favoring radiation resistance [132] or immune evasion [133]. ICAM-5 seems to play a role in tumorigenesis and perineural invasion through the PI3K/Akt pathway as well. Indeed, Maruya and colleagues [134] observed a high incidence of perineural invasion in ICAM-5 rich specimen and a decreased ICAM-5 expression following PI3K inhibition.

### VCAM-1

VCAM-1 has been reported to be expressed by numerous tumor cell types of pancreatic, breast and gastric cancers [135–138]. This expression enables the interaction of cancer cells with cancer associated macrophages harboring α4-integrins leading to tumor growth support [136]. Similar phenomena were observed in glioblastoma where exposure to IL-1β induced the expression of VCAM-1 and ICAM-1 on tumor cells. Their presence allowed the adhesion and polarization of tumor-associated monocytes [139]. In pancreatic cancer, lactate production induced by VCAM-1 from pancreatic cancer cells with enhanced aerobic glycolysis activated macrophages to a TAM-like phenotype [135]. The contribution of VCAM-1 to metastasis is well described in literature [77]. CXCL1 and CXCL13 are able to induce VCAM-1 expression in osteosarcoma cells through the NF-kB pathway, which in turns favors VCAM-1 dependent migration of cells [140,141]. In a melanoma model, Klemke et al. [142] demonstrated that the migration of cancers cell lines was dependent on the VLA-4 and VCAM-1 interaction at the endothelial level. Authors also pointed out that the affinity between these two molecules was positively correlated to the aggressiveness of the cancer cell line. Investigating the specialized environment of the brain, Sikpa et al. [143] deciphered the implication of VCAM-1 in the formation of brain metastasis. Upregulation of VCAM-1 in the vessels following cerebrovascular inflammation promoted the interaction of circulating tumor cells with endothelial cells and thus their extravasation. Inflammation also drove lymphatic permeability and invasion by cancer cells when VCAM-1 was induced in lymphatic endothelial cells [144]. Regarding vascular permeability, M2 macrophages seemed to contribute to vascular permeability via the VCAM1/RAC1/ROS/p-PYK2/p-VE-cadherin cascade initiated by interaction between VLA4 and VCAM-1 on endothelial cells in ovarian cancer [145]. Not only surface VCAM-1, but also secreted VCAM-1 seems to promote tumor progression. Indeed, cancer associated fibroblasts are able to secrete VCAM-1 which in turns increase growth and invasion through the AKT and MAPK pathways in lung cancer cells [146]. The expression of VCAM-1 is closely related to angiogenesis and VEGF secretion by tumor cells although their relative influence to each other remains complex. Sustained levels of VEGF correlated with low levels of VCAM-1 in the endothelium that may consist in a mechanism of defense from tumor cells promoting their immune evasion by precluding immune infiltration [147,148]. On the other hand, studies have reported areas of high microvessel density that correlated with high VCAM-1 expression in various cancers [137,149]. Sano et al [150] observed a reduction of tumor angiogenesis which led to a decreased tumor growth and metastasis in presence of endothelial VCAM-1 inhibition. In human samples, the use of VCAM-1 as prognostic marker has grown in interest. In the plasma of preoperative patients of urothelial bladder carcinoma, elevated level of VCAM-1 was associated with aggressive features such as lymph-node-metastasis or ≥pT3 disease while no correlation with overall survival (OS) or progression free survival (PFS) could be assessed [151]. In the tumor tissue of nasopharyngeal carcinomas, VCAM-1 was associated with chemotherapy resistance, shorter progression free (PFS) and overall survival (OS) [152]. Additionally, high levels of soluble VCAM-1 were also associated with shorter PFS and OS in advanced breast cancer patients [153]. Conversely, in metastatic colorectal cancer, soluble VCAM-1 appears to improve OS benefit in the context of a regorafenib treatment [154].

## Impact of endothelial adhesion molecules on anti-tumor immunity in the context of immunotherapies

Adhesion molecules also harbor an anti-tumor role by re-shaping the tumor immune microenvironment. This role could be of increasing importance particularly in the context of immunotherapies.

### Selectins

While selectins expressed at distant locations from the tumor can participate to the invasion of target tissues by circulating tumor cells, the expression of E-Selectin ligands by the immune cells may have a determinant role in preventing tumor progression [155]. Endothelial P and L-Selectin inhibition in a model of colon carcinoma and melanoma was associated to enhanced tumor growth, which may be related to the lack of monocyte infiltration within tumors [156]. The importance of selectins in cancer immunity have been further confirmed by Stark et al. [157]. In their study, the contribution of selectins in E/P/L-selectin deficient mice was highlighted: compared to control mice, the infiltration of CD8+ T cells into the draining lymph nodes and tumors was impaired thus resulting in significantly shorter survival. Weishaupt et al [158], suggested that the induction of endothelial E-Selectin and ICAM-1 was essential to improve tumor control in metastatic melanoma. For treatments involving CAR-T-cells, the expression of E-Selectin at the target site was shown to be mandatory for CAR T-cells to reach the target for effectiveness [159]. While the activation status of lymphocytes is critical for tumor control, the functional homing of effector lymphocytes promoted by selectins is also required. Indeed, Aires et al, showed that selectin ligand-deficient mice were not able to limit tumor growth when compared to controls [160]. Interestingly, these differences were unrelated to antigen recognition or effector T-cell function, pointing out an important role for selectins in getting the effector cells in the right location.

Among selectins, L-Selectin has been described as a major molecule for the development of an immunity directed against tumors. Indeed, L-Selectin facilitates lymphocyte homing to lymph nodes (LN). L-Selectin blockade prevented the homing of lymphocytes in lymph nodes and primary tumor sites, thus impairing the development of a specific cytotoxic T cell response [161]. Myeloid derived suppressor cells (MDSC) were able to target lymphocytes homing to lymph nodes by cleaving L-selectin expressed by T-cells with surface ADAM17 which resulted in a decreased antigen-specific response [162,163]. Consequently, the targeting of MDSC with doxorubicin improved the killing efficacy of cytotoxic lymphocytes. The elimination of tumors by lymphocytes was antigen-specific and resulted from an upregulation of CD3ζ and L-selectin in cytotoxic T-cells [164]. L-Selectin has also been investigated in the context of immunotherapy. Watson et al. [165] found that mice deficient for L-Selectin showed faster tumor progression. Interestingly, mice with persistent L-Selectin expression at the surface of T cells had a reduced tumor growth. Tsui et al [166] focused on exhausted T-cells and restoration of function after PD1 blockade. They identified a subpopulation of exhausted T cells specifically expressing L-Selectin that exclusively proliferated in response to anti-PD-1 therapy. Finally, Kumari et al. [167] confirmed the implication of L-Selectin expressed by B-cells and T-cells with the positive outcomes in breast cancer samples from patients. They found a strong correlation between the SELL gene and a pro-inflammatory tumor microenvironment, including B- T-cells and M1 macrophages. High SELL expression was associated with favorable survival in breast cancer as well. These observations were later confirmed in colorectal cancer patient samples and in breast when SELL was upregulated in tumor tissues [168].

### ICAM

The crucial role of ICAM-1 in limiting tumor progression was reported by several studies. The depletion of ICAM-1 in T-cells led to a lack of immune infiltration in tumors with poor cancer control [169–171]. It had been observed in breast cancer that patients with elevated ICAM-1 tumor levels had a better survival, Regev et al found that while ICAM-1 deletion did not affect primary breast tumor growth, there was an increase in spontaneous metastasis to the lungs in ICAM-1 KO models. The control of lung metastasis was mediated by neutrophils, binding ICAM-1 expressed in the lung vasculature [172]. Figenschau et al highlighted ICAM-1 expression by cancer cells was associated with tertiary lymphoid structure formation within tumors [173]. In malignant melanoma, the primary and metastatic tumor control were affected by the loss of L-Selectin and/or ICAM-1 on tumor cells [174]. This phenotype was associated with a general depletion in the infiltrating populations including natural killer (NK) cells, CD4+ and CD8+ T cells, but also pro-inflammatory cytokines such as IFN-γ or TNF-α. The absence of ICAM-1 may also contribute to the resistance of cancer cells by precluding specific immune cells to interact with the tumor tissue. Indeed, Liu et al. [175] reported pancreatic tumor cells to be resistant to γδ-T-cells because of the poor binding occurring in absence of ICAM-1 or ICAM-2 at the surface of the cancer cells. The transfection of resistant cells with ICAM-1 or ICAM-2 subsequently restored the sensitivity of pancreatic tumor cells to γδ-T-cells. Interactions between immune cells and cancer cells through ICAM-1 are also responsible for intratumoral retention of activated CD8+ T-cells. As demonstrated by Yanguas et al. [176] the blocking of ICAM-1 in the tumor reduced the clusters of lymphocytes inside the tumors by allowing their homing to LN. Yang et al. [177] observed that ICAM-1 expression in the tumor inversely correlated with macrophage infiltration while deficiency in ICAM-1 resulted in the specific increase in M2 macrophages population. Interestingly, the increase in M2 subpopulation results from a polarization of macrophages toward this phenotype that appeared to be enhanced by an increase of efferocytosis of apoptotic cells through the PI3K/AKT pathway.

The impact of ICAM-1 on the response to immunotherapy remains a matter of debate. The soluble fraction of ICAM-1 in the blood of hepatocellular carcinoma patients correlated positively with a better OS and a lower recurrence rate but was also predictive of a poor response to immune checkpoint inhibition [178]. However, Taggart et al observed an increase in immune populations following combined CTLA-4/PD-1 treatment. An upregulation of ICAM-1 and VCAM-1 in the tumor following treatment appeared to be responsible for this phenotype [179]. This relation was also reported by Schneider et al, who observed an increase in ICAM-1 binding following anti-CTLA-4 exposition [180]. In combination with bevacizumab, anti-CTLA-4 enhanced tumor infiltration by promoting E-Selectin, ICAM-1 and VCAM-1 at the surface of melanoma cells [181]. In relation with the CAR T-cells field, ICAM-1 at the surface of tumor cells was reported as a good target for CAR T-cells in reason of its wide expression across tumors [182]. Interestingly, PD1 blockade in combination with ICAM-directed CAR T-cell enhanced tumor cells eradication compared to CAR T-cell alone, suggesting a synergic effect [183]. In addition, the expression of ICAM-1 at the surface of Ewing’s sarcoma following exposure to pro-inflammatory cytokines improved the recognition and killing of the tumor cells by specific CAR T-cell [184].

Of note, ICAM-2 is specifically reported as increasing anti-tumor immunity. Transduction of ICAM-2 by intratumoral injection significantly inhibited tumor growth in subcutaneous gastric tumors. The reduction of tumors growth was associated with an increase in NK cells infiltration [185]. ICAM-2 also promoted survival of immune cells. Through the activation of PI3K/AKT pathway, ICAM-2 precluded CD19+ cells from apoptosis [186].

### VCAM-1

VCAM-1 endothelial expression, as the previous adhesion molecules, exerts its anti-tumoral contribution through the recruitment of immune cells directed against cancer. In tumors, endothelial cells are known to be anergic and not efficiently promote immune infiltration. In an attempt to restore immune response inside the tumors, Nakajima et al. [187] treated pancreatic tumors cell with Embelin to increase levels of VCAM-1 and E-Selectin in endothelial cells within tumors. In response to this upregulation, authors noted a higher infiltration of anti-tumor immune cells, which ultimately led to an improved tumor control. Conversely, following inhibition, Sasaki et al highlighted the crucial role of VLA-4/VCAM-1 interaction in immune infiltration of tumors. More specifically, Th1 T-cell infiltration was restricted when VCAM-1 at their surface was abrogated, leading to a loss of immune mediated tumor control [188,189]. Campisi et al showed that cGAS-STING signaling from tumor cells ultimately increased the expression of E-Selectin, ICAM-1 and VCAM-1 on tumor endothelium, thus enhancing immune cell extravasation [190].

**Figure 2.**
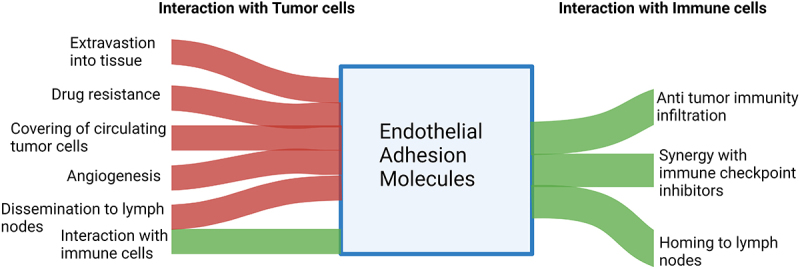
Modified Sankey plot depicting the contribution of endothelial adhesion molecules interaction with tumor cells and immune cells. Positive contributions are represented in green while negative contributions are colored in red.

VCAM-1 appears to enhance the response to immunotherapy. Riegler et al. [191] showed that endothelial VCAM-1 was an interesting in vivo predictor of both immune infiltration and response to immunotherapy in a preclinical model of MC38 tumors. Endothelial VCAM-1 density obtained by noninvasive imaging correlated with tumor infiltration and response to PDL-1 blockade. Moreover, blocking interaction between T-cell and VCAM precluded tumor rejection. Studies on patients also support the beneficial effect of VCAM-1 in checkpoint blockade. In NSCLC, high serum levels of VCAM-1 correlated with an improved OS in patients treated with second line nivolumab [192]. In the context of cancer vaccination, the combination of sunitinib and peptic-pulsed dendritic vaccines displayed the best results in tumor regression compared to control groups. It appears sunitinib contributed to increase the expression of VCAM-1 at the surface of vascular cells, allowing an improved recruitment of vaccination-induced cytotoxic T-cell [193]. Results are similar to Garbi et al. [194] who treated pancreatic islet cell carcinomas with antigen-specific vaccination (Tag) and vaccination to oligodeoxynucleotides (ODN) with cytosine-guanine-rich (CpG) motifs (CpG-ODN) to enhance immunity generated by Tag. Interestingly, while Tag successfully primed T-cell, immune cells were not able to penetrate into the tumor tissue. CpG-ODN concomitant exposition acted as a pro-inflammatory agent which upregulated ICAM-1 and VCAM-1 at the surface of vascular cells. This systemic stimulation ultimately enhanced the extravasation of primed T-cells, which translated into an improved tumor control. Both pro-tumoral and anti-tumoral impacts of adhesion molecules are recapitulated in Table 1. The contribution of endothelial adhesion molecules for their interaction with tumor and immune cells is resumed in Figure 2.

**Table 1.** Contribution of adhesion molecules in tumor progression and antitumor response establishment.

Adhesion molecules	Pro-tumor role	Anti-tumor role	Models used
E-Selectin	Metastasis promotion by extravasation into tissue [88–90] Drug resistance through NF-kB and Wnt pathway [92–95]	Anti-tumor immunity infiltration [156,157,160]	Pro-tumor role: Murine cancer cell lines: Liver [88], various [92], breast [95]; Human cancer cell lines: Liver [88], various [89], breast [90], acute myeloide leukemia [93,94] Anti-tumor role: Murine cancer cell lines: Melanoma [157], plasmacytoma [160]; Human cancer cell line: Colon [156], melanoma [156]
P-Selectin	Metastasis promotion by extravasation into tissue [90,103] Covering of circulating tumor cells in systemic circulation [98,99] Cancer-associated thrombosis [107–109]	Anti-tumor immunity infiltration [157,160]	Pro-tumor role: Murine cancer cell lines: leukemia [103], colon carcinoma [99]; Human cancer cell lines: Breast [90,107], acute lymphoblastic leukemia [103], colon carcinoma [98], various [108,109] Anti-tumor role: Murine cancer cell lines: Melanoma [157], plasmacytoma [160]
L-Selectin	Metastasis promotion by extravasation into tissue [90,103,110,112,113] Dissemination to tissue and lymphatic system [111,114]	Anti-tumor immunity infiltration [157,160] Homing of lymphocytes to lymph nodes [162,163] Synergic effect with immune checkpoint inhibitors [165,166]	Pro-tumor role: Murine cancer cell lines: leukemia [103], insulinoma [111], colon carcinoma [112,113]; Human cancer cell lines: Breast [90,107], acute lymphoblastic leukemia [103,110], various [113], head and neck [114] Anti-tumor role: Murine cancer cell lines: Melanoma [157,165], plasmacytoma [160], breast [162], various [163], chronic infection [166]
ICAM	Metastasis promotion by extravasation into tissue [115–117] Cancer angiogenesis [121–123] Drug resistance (ICAM-3) [132] Immune evasion (ICAM-3) [133]	Anti-tumor immunity infiltration [169–171] Formation of tertiary lymphoid structure [173] Effective target for CAR T-cells [182]	Pro-tumor role: Murine cancer cell lines: Melanoma [115]; Human cancer cell lines: Melanoma [115,117], breast [116,121], colon carcinoma [122], lung [123], various [132], gastric cancer [133] Anti-tumor role: Murine cancer cell lines: virus induced tumors [169], various [170,182]; Human cancer cell line: colon carcinoma [171], breast [173]
VCAM-1	Mediates interaction between cancer cells and tumor associated macrophages [136,139] Metastasis promotion by extravasation into tissue [77,142] Cancer angiogenesis [137]	Anti-tumor immunity infiltration [147,148,187–189] Synergic effect with immune checkpoint inhibitors [191,192]	Pro-tumor role: Murine cancer cell lines: Glioblastoma [139]; Human cancer cell lines: Breast [136], glioblastoma [139], various [77], melanoma [142], gastric cancer [137] Anti-tumor role: Murine cancer cell lines: Melanoma [147,188,189], breast [148], lung [148], various [191]; Human cancer cell line: Colon carcinoma [147], breast [148], pancreatic ductal adenocarcinoma [187], lung [192]

Overall, data from the literature suggests that adhesion molecules such as E-Selectin, ICAM-1 and VCAM-1 can either favor (through vascular tumor cell intravasation) or limit (through enhanced tumor immune infiltration and efficient immune response development) tumor growth, Table 2. Changes in the expression of ICAM-1 at the surface of newly formed vessels can affect T-cell infiltration. Several studies reported the emergence of poorly perfused and permeable vessels following expression of pro-angiogenic factors in tumors [195,196]. At the surface of those aberrant vessels, clustering defect of ICAM-1 and VCAM-1 induced by VEGF-A are partially responsible for the hampering of immune cell infiltration [197]. In addition, it has been reported that VEGF reduces the expression of ICAM-1, E-Selectin and VCAM-1 at the surface of endothelial cells [198,199]. Therefore, treatments aiming to relieve the vascular anergy in the aberrant tumor vasculature could enhance immune cells infiltration through adhesion molecule expression.

**Table 2.** Summary of human studies investigating the prognosis value of adhesion molecules. OS: overall survival, NSCLC, non-small cell lung cancer.

Name of first author	Number of patients	Cancer type	Main finding
Tanio et al. [85]	117	Renal cancer	Membrane expression of functional E-selectin correlated more significantly with poor prognosis of patients
Chien et al. [87]	89	AML	E-selectin ligand expression in blast correlates with lower survival and higher propagation
Aref et al. (2002) [110]	50	AML	Patients with higher soluble E and L selectins levels had shorter event free surviva than patients with lower levels.
Papachristos et al. [122]	46	Colorectal cancer	The ICAM-1 rs1799969 G/A allele was associated with prolonged OS.
Maeda et al. [125]	96	Colorectal cancer	Incidence of lymph node or liver metastasis was significantly lower in patients with ICAM-1-positive tumors
Liu et al. [154]	149	Colorectal cancer	Soluble VCAM-1 was also potentially predictive of lower OS and of benefit from regorafenib
Li et al. [168]	613	Colorectal cancer	SELL expression was associated with favorable outcomes in CRC patients
Dowlati et al. [123]	878	NSCLC	Patients with low baseline soluble ICAM had a higher response rate better overall survival better 1-year survival (65% versus 25%) than those with high ICAM
Carbone et al. [192]	71	NSCLC	High baseline serum levels of VCAM-1 are associated with a longer survival in patients treated with nivolumab as second line treatment for NSCLC
Jung et al. [126]	157	Gastric cancer	Increased expression of intercellular adhesion molecule-1 in gastric cancer could be related to the aggressive nature of the tumor, and has a poor prognostic effect on gastric cancer
Liu et al. [133]	504	Gastric cancer	ICAM-3 expression in tumor is associated with immune evasion
Ding et a.l [137]	41	Gastric cancer	VCAM-1 positive cancers were associated with more lymph node metastases than VCAM-1-negative ones
Byrne et al. [149]	93	Breast cancer	Women who developed early recurrence had higher preoperative levels of serum VCAM-1 than those who remained disease free
Schröeder et al. [127]	169	Breast cancer	ICAM-1 expression in the tumor was associated with a more aggressive tumor phenotype
Kumari et al. [167]	77	Breast cancer	SELL expression is associated with favorable survival outcomes
Bulska-Będkowska et al. [153]	39	Breast cancer	Higher levels of soluble ICAM-1 were associated with faster progression of breast cancer
Mori et al. [151]	1036	Urothelial carcinoma	Preoperative plasma VCAM-1 was significantly elevated in patients with adverse pathologic features. Higher VCAM-1 levels were independently associated with increased risk of lymph-node-metastasis, ≥pT3 disease, and non-organ-confined disease and lower recurrence-free survival, cancer-specific survival, and overall survival (OS) in pre- and postoperative multivariable models
Huang et al. [152]	73	Nasopharyngeal carcinoma	Patients with high VCAM-1 expression were more prone to shorter periods of PFS and OS
Cao et al. [178]	87	Hepatocarcinoma	Patients with elevated level of soluble ICAM-1 showed the lowest TFS and OS but higher immune cells count
Wu et al. [181]	43	Melanoma	Expression of E-selectin and VCAM1 on melanoma tumor-associated endothelial cells promoted adhesion of activated T cells onto endothelial cells

## Summary: selectins, ICAM-1, VCAM-1: pro or anti-tumoral role?

In light of this review, adhesion molecules have a broad impact on tumors and their microenvironment. Therefore, their study and therapeutic activation/inhibition should be balanced with the perspective that they can promote (metastasis development) or inhibit (anti-tumor immune microenvironment remodeling) cancer progression. Their study should thus take into consideration the location of expression, the cancer type, its immune microenvironment and metastatic potential.

Specifically, E-Selectin tumor or distant site endothelial expression promotes cancer progression via chemoresistance [93,94] and cancer cell extravasation in distant organs respectively [89,90]. Conversely, E-Selectin expression in endothelial cells at the primary tumor site promotes better local control because of better immune cell infiltration [156,159]. The impact of L-Selectin was similar although cancer dissemination was mostly promoted via lymphatics [111,114,162,164]. Finally, tumor P-Selectin mostly had a tumor dissemination role by protecting, via platelet aggregation, cancer cells from blood stream shear stress [98,99].

The impact of ICAM-1 expression seems more ambivalent. When expressed on tumor cells, ICAM-1 harbors a pro-tumoral role and promotes metastasis [118,119]. However, this same tumor expression favors a local response against the tumor by tertiary lymphoid structure formation inside tumors [173,176]. Therefore, its inhibition requires a contextualization of the tumor type and immune microenvironment. Moreover, ICAM-1 is an effective target for tumor inhibition following CTLA-4 abrogation or CAR-T cells treatment [180,183].

Finally, VCAM-1 expression on tumor cells contributes mostly to cancer progression via the recruitment of macrophages and subsequent changes in tumor vasculature favoring cancer cell dissemination [135,139,145]. However, VCAM-1 expression on endothelial cells favors a change in the immune microenvironment promoting T-cell infiltration of tumors and cancer control [187–189].

In conclusion, contribution of adhesion molecules to tumor progression depends on the location of expression, the cancer type and immune microenvironment and potential associated therapies. Further studies in different cancer contexts are required to highlight the contribution of adhesion molecules for cancer control of progression.

## Conclusion

The endothelium plays a critical role in tissue homeostasis by regulating their interaction with circulating elements. Adhesion molecules at their surface are hijacked by tumor cells to enter or exit the circulation during the metastasis process. Conversely, these same adhesion molecules are essential for patients to develop an anti-tumor immunity through the recruitment and elaboration of a cytotoxic immune response. Therefore the therapeutic manipulation of adhesion molecules requires attention because of their ambivalent role and should be tailored to the cancer type and its immune microenvironment. Based on this review, a specific focus on expression of L-Selectin and VCAM-1 could have a promising anti-tumoral effect through their capacity to enhance anti-tumor immunity alone or in combination with immunotherapy while not being so involved in cancer cell dissemination. Further research in this field is mandatory.

## Data Availability

Data sharing is not applicable to this article as no new data were created or analyzed in this study.
